# Fracture Risk in Men with Metastatic Prostate Cancer Treated With Radium-223

**DOI:** 10.1016/j.clgc.2021.03.020

**Published:** 2021-10

**Authors:** Adham Hijab, Sebastian Curcean, Nina Tunariu, Holly Tovey, Roberto Alonzi, John Staffurth, Matthew Blackledge, Anwar Padhani, Alison Tree, Helen Stidwill, Jessica Finch, Peter Chatfield, Sophie Perry, Dow Mu Koh, Emma Hall, Chris Parker

**Affiliations:** 1 The Royal Marsden NHS Foundation Trust, London, UK; 2 The Institute of Cancer Research, London, UK; 3 Mount Vernon Cancer Centre, Northwood, UK; 4 Velindre Cancer Centre, Cardiff, UK; 5 Paul Strickland Scanner Centre, Mount Vernon Cancer Centre, Northwood, UK

**Keywords:** Skeletal-related events, Bone health, Metastatic castration-resistant prostate cancer, Bone metastasis

## Abstract

**Background:**

Radium-223 is a bone-seeking, alpha-emitting radionuclide used in metastatic castration-resistant prostate cancer (mCRPC). Radium-223 increases the risk of fracture when used in combination with abiraterone and prednisolone. The risk of fracture in men receiving radium-223 monotherapy is unclear.

**Patients and Methods:**

This was a prospective, multicenter phase II study of radium-223 in 36 men with mCRPC and a reference cohort (*n* = 36) matched for fracture risk and not treated with radium-223. Bone fractures were assessed using whole-body magnetic resonance imaging. The primary outcome was risk of new fractures.

**Results:**

Thirty-six patients were treated with up to six 4-week cycles of radium-223. With a median follow-up of 16.3 months, 74 new fractures were identified in 20 patients. Freedom from fracture was 56% (95% confidence interval, 35.3-71.6) at 12 months. On multivariate analysis, prior corticosteroid use was associated with risk of fracture. In the reference cohort (*n* = 36), 16 new fractures were identified in 12 patients over a median follow-up of 24 months. Across both cohorts, 67% of all fractures occurred at uninvolved bone.

**Conclusions:**

Men with mCRPC, and particularly those treated with radium-223, are at risk of fracture. They should receive a bone health agent to reduce the risk of fragility fractures.

## Micro-Abstract

Radium-223 increases the risk of fracture in combination with abiraterone and prednisolone. The fracture risk of radium-223 monotherapy is unclear. This prospective study assessed fracture incidence in men treated with radium-223 matched with a cohort that did not have radium-223. We identified 74 new fractures in 20 patients receiving radium-223. Most fractures occurred in bones without apparent metastases. Bone health agents should be mandatory before starting radium-223.

## Introduction

Radium-223 is a bone-seeking, alpha-emitting radionuclide that is approved for use in men with metastatic castration-resistant prostate cancer (mCRPC). The ALSYMPCA trial showed that radium-223 improves both overall survival and time to skeletal-related events.^1^ Subsequently, the ERA 223 trial, which compared abiraterone and prednisolone plus radium-223 versus abiraterone and prednisolone plus placebo, found a substantially increased risk of fracture in patients randomized to abiraterone and prednisolone plus radium-223.^2^ In ALSYMPCA, the proportion of patients reporting a pathologic fracture was 4% for radium-223 and 5% for placebo. In the ERA 223 trial, the risk of fracture within 12 months was 23% for abiraterone and prednisolone plus radium-223 compared with 7% for abiraterone and prednisolone plus placebo. The explanation for the contrasting results of these two trials remains uncertain.

One hypothesis is that radium-223 increases the risk of fracture only if used in combination with other agents that have an adverse effect on bone health, such as abiraterone and prednisolone.^3^ An alternative explanation relates to the frequency of imaging in the two trials: In ALSYMPCA, imaging was done according to clinical need and not mandated at regular intervals. In ERA 223, computed tomography (CT) and bone scans were done every 3 months. It is possible that radium-223 increases the risk of fracture, even when used as a single agent, but that this was not observed in ALSYMPCA because of the lack of routine imaging.

We have conducted a prospective phase II study (REASURE) of radium-223 used as a single agent, incorporating routine whole-body magnetic resonance imaging (WB-MRI), in men with mCRPC. The primary objective of the study was to evaluate treatment response by whole-body diffusion-weighted MRI.^4^ Here, we present an exploratory analysis of the risk of fracture during and after treatment. The objective of this exploratory analysis was to describe the risk of fracture in men receiving radium-223 as a single agent. We also assessed, as a benchmark, the fracture risk in a similar cohort of men with mCRPC who were imaged in the same way but did not receive radium-223.

## Methods

Patients with chemotherapy-naïve, bone-only, progressive mCRPC were enrolled in a prospective study of radium-223. They received treatment with radium-223 every 4 weeks for up to six cycles. Patients were randomized to one of two dose-levels: 55 or 88 kBq/kg. WB-MRI scans were done at baseline, at cycles 2 and 4, and at 1 month after treatment. During the follow-up period, patients were evaluated every 4 months for 1 year, following which imaging was done according to routine clinical practice and scans were collected and reviewed centrally. The WB-MRI protocol included standard T1 weighted (T1w) and T2w sagittal spine supplemented by axial T1w Dixon, T2w HASTE, and diffusion-weighted imaging (DWI) sequences. The presence of a fracture was determined on T1w and T2w sequences; differentiation between a malignant cause and non-malignant cause (presumed osteoporotic) was accomplished by using all available sequences, including DWI and the T1 Dixon-derived fat fraction.^5^, ^6^, ^7^, ^8^, ^9^

The REASURE trial was registered (ISRCTN17805587), approved by the National Research Ethics Service London–Surrey Borders Research Ethics Committee (14/LO/1385), co-sponsored by the Institute of Cancer Research and The Royal Marsden NHS Foundation Trust, and conducted in accordance with the principles of good clinical practice. All of the participants provided written informed consent prior to study entry. The Clinical Trials and Statistics Unit at the Institute of Cancer Research (London, UK) coordinated the study.

We also identified a separate reference cohort of men with mCRPC treated at The Royal Marsden Hospital but not treated with radium-223 to act as a benchmark for the fracture rate. This cohort was used in order to provide an estimate of the fracture rates among patients with similar disease pattern, utilization of bone-health agents, and corticosteroid usage compared with the REASURE population. Moreover, the extracted cohort also had regular use of WB-MRI during follow-up. Scans were done as part of routine clinical practice, with a median time between scans of approximately 5 months. The use of this reference cohort was approved by the Committee for Clinical Research of the Institute of Cancer Research and The Royal Marsden Hospital.

The scans were reviewed by a radiologist experienced in mCRPC. Fractures were identified on whole body MRI in correlation with all other imaging available and the underlying bone was assessed as malignant or uninvolved depending on MRI appearances.

### Statistical Methods

Fractures identified prior to starting treatment were not included in analyses. All subsequent imaging tests were used for fracture assessment. Time to new fracture was calculated from the date of randomization in REASURE to the first scan showing a new fracture. Time to fracture was presented using Kaplan–Meier curves and association with other variables assessed via log-rank tests and Cox proportional hazards models. The reference cohort was used as a benchmark only; no formal comparisons were made between the REASURE trial population and the reference cohort.

## Results

Between July 2015 and June 2017, 39 patients were randomized in a 1:1 ratio between the two radium-223 dose levels. Three patients were excluded from analysis of fractures: two due to unmet eligibility criteria and one due to poor MRI tolerance, leaving 36 evaluable patients. In the evaluable population, median age was 75 years (interquartile range [IQR], 72-80). Baseline patient characteristics are provided in Table 1. Of note, bone health agents were used in only four patients (11%). Eight patients (22%) were previously treated with abiraterone or enzalutamide. Patients were followed up for a median of 16.3 months (IQR, 5.8-26.1).

**Table 1:** Baseline Patient Characteristics.

		REASURE Cohort						Reference Cohort	
		55 kBq/kg (*n* = 19)		88 kBq/kg (*n* = 17)		Total (*N* = 36)		Total (*N* = 36)	
		*n*	%	*n*	%	*n*	%	*n*	%
Age (y), median (IQR)		75.6 (73.0-80.1)		74.5 (72.6-78.1)		75.1 (72.8-79.5)		70.6 (63.1-73.9)	
Weight (kg)^a^									
	Median (IQR)	80.0 (70.0-91.0)		79.3 (70.6-101.6)		79.7 (70.2-91.3)		82.0 (73.0-92.0)	
	<80	9	47.4	9	52.9	18	50.0	17	47.2
	≥80	10	52.6	8	47.1	18	50.0	19	52.8
ALP (U/L)^a^									
	Median (IQR)	99.0 (73.0 – 235.0)		106.0 (94.0 – 128.0)		105.0 (83.5 – 174.5)		86.5 (65.0 – 152.5)	
	<220	13	68.4	16	94.1	29	80.6	31	86.1
	≥220	6	31.6	1	5.9	7	19.4	5	13.9
Bisphosphonate use^a^									
	Yes	3	15.8	1	5.9	4	11.1	2	5.6
	No	16	84.2	16	94.1	32	88.9	34	94.4
Extent of bone disease									
	<6 metastases	9	47.4	8	47.1	17	47.2	17	47.2
	6-20 metastases	10	52.6	9	52.9	19	52.8	19	52.8
Prior corticosteroids									
	No	8	42.1	10	58.8	18	50.0	18	50.0
	Yes	11	57.9	7	41.2	18	50.0	18	50.0
Had fractures at baseline									
	No	17	89.5	15	88.2	32	88.9	32	88.9
	Yes	2	10.5	2	11.8	4	11.1	4	11.1

a Stratification factors at randomization (REASURE cohort). Abbreviations: ALP = alkaline phosphatase; IQR = interquartile range.

Out of 36 evaluable patients, 20 patients (56%) completed six cycles of radium-223, and the remaining 16 patients discontinued early due to disease progression. The median number of treatment cycles among patients who discontinued early was four (Table 2).

**Table 2:** Treatment Compliance (REASURE Cohort).

		55 kBq/kg (*n* = 19)		88 kBq/kg (*n* = 17)		Total (*N* = 36)	
		*n*	%	*n*	%	*n*	%
Completed 6 cycles		10	52.6	10	58.8	20	55.6
Discontinued early		9	47.4	7	41.2	16	44.4
Number of cycles completed if discontinued early							
	3	4	21.1	3	17.6	7	19.4
	4	4	21.1	3	17.6	7	19.4
	5	1	5.3	1	5.9	2	5.6

**Figure 1:**
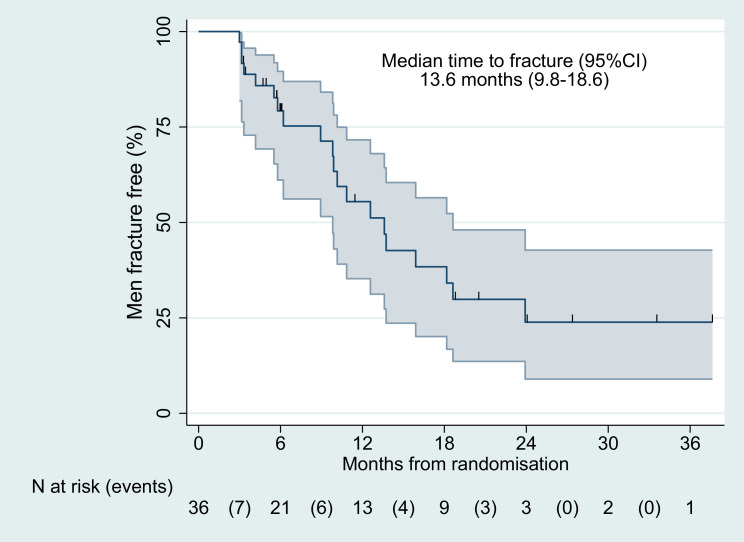
Kaplan–Meier Curve for the REASURE Trial Population. Abbreviation: CI = confidence interval.

**Figure 2:**
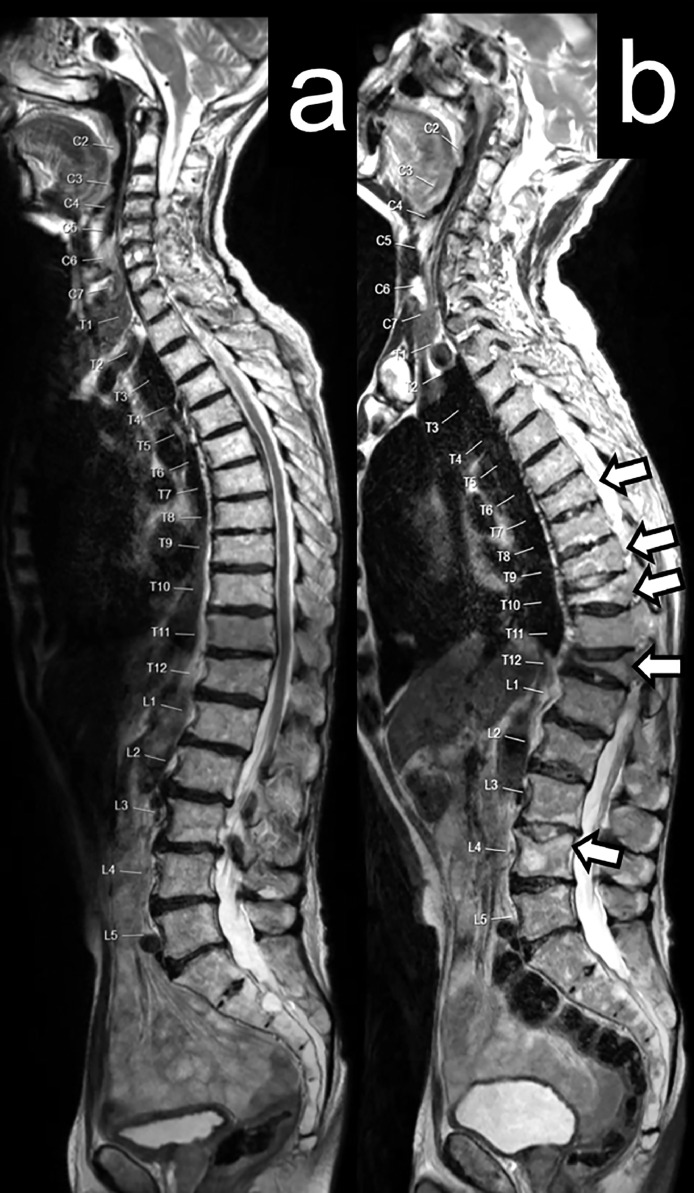
MRI Images Showing New Fractures in a REASURE Patient. Sagittal T2w Composed Spine Images in a 71-Year-Old Man With mCRPC at Baseline (a) and 2 Years After Six Cycles of Radium-223 (b). Follow-Up Images Show Multiple Non-Malignant Endplate Vertebral Fractures at T7, T8, T9, T10, T12, and L4 (White Arrows) With Significant Vertebral Collapse at T10 and T12. Abbreviation: MRI = magnetic resonance imaging; mCRPC = metastatic castration-resistant prostate cancer.

In total, 205 imaging scans after starting radium-223 were evaluated. Overall, 74 new fractures were identified in 20 patients (56%). Freedom from fracture was 79% (95% confidence interval [CI], 61.1-89.6) at 6 months and 56% (95% CI, 35.3-71.6) at 12 months (Figure 1). Median time to first new fracture was 13.6 months (95% CI, 9.8-18.6). Most new fractures developed within the axial skeleton (Table 3). Out of all fractures, 50 fractures (68%) occurred at a site of uninvolved bone (Table 3; see example case in Figure 2). Of the 20 patients who had new fractures, 10 patients (50%) had symptomatic fractures, four had asymptomatic fractures, and six had uncertain symptomatic status.

**Table 3:** Fracture Location and Distribution Based on Bone Status (REASURE Cohort) and Metastasis Involvement.

Location	Patients, *n* (%)	New Fractures, *n*	New Fractures at Site of Metastasis, *n*
Spine	14 (38.9)	49	15
Thorax	7 (19.4)	19	4
Pelvis	4 (11.1)	5	4
Extremities	1 (2.8)	1	1
Total	20 (55.6)	74	24

On univariate analysis, high disease burden and baseline alkaline phosphatase (ALP) were associated with risk of fracture. There was no significant association between radium-223 dose and risk of fracture (Table 4). On multivariate analysis, prior corticosteroid use was the only variable significantly associated with risk of fracture.

**Table 4:** Fracture Risk in REASURE Trial Population by Cox Regression Analysis

		Univariate Model			Multivariable Model		
		Hazard Ratio	95% CI	*P*	Hazard Ratio	95% CI	*P*
Treatment (kBq/kg)							
	55	1	—	.949	1	—	.728
	88	0.97	0.40-2.35		0.81	0.25-2.66	
Extent of bone disease							
	<6 metastases	1	—	.027	1	—	.194
	≥6 metastases	3.00	1.12-8.04		2.21	0.66-7.37	
Weight (kg)							
	<80	1	—	.063	1	—	.249
	≥80	0.42	0.17-1.05		0.53	0.18-1.56	
ALP (U/L)							
	<220	1	—	.040	1	—	.343
	≥220	5.92	1.22-28.74		2.58	0.37-18.09	
Bisphosphonates							
	Yes	1	—	.710	1	—	.981
	No	0.78	0.23-2.72		1.02	0.20-5.08	
Prior corticosteroids							
	No	1	—	.069	1	—	.042
	Yes	2.3	0.93-5.7		3.06	1.03-9.11	

Estimates from the multivariable model were adjusted for treatment dose, extent of disease, weight, ALP levels, bisphosphonates use, time since diagnosis, time since bone metastases, cycles of radium, and prior corticosteroid use.

Abbreviations: ALP = alkaline phosphatase; CI = confidence interval.

Median age for the reference cohort (*n* = 36) was 70 years (IQR, 63-74). Thirty-one patients (87%) were starting treatment with abiraterone or enzalutamide, 18 patients (50%) had prior corticosteroids, and two patients (6%) received bone health agents. The median follow-up time for the benchmarking group was 24.0 months (IQR, 17.1-26.6). In total, 150 imaging scans were evaluated. Overall, 16 new fractures were identified in 12 patients (33%). Out of all fractures, 10 fractures (62%) occurred at a site of uninvolved bone.

## Discussion

We observed a fracture risk at 1 year of 44% in men with mCRPC treated with radium-223 as a single agent. Most fractures were of uninvolved bone, not at the site of metastases. These data suggest that men with mCRPC receiving radium-223 are at high risk of fracture and highlight the role of bone health agents for the prevention of fragility fractures in these patients.

It was already known that the use of radium-223, in common with other treatments for mCRPC,^3^^,^^10^ increases the risk of fracture when used in combination with abiraterone and prednisolone.^2^ However, there has been a lack of evidence until now concerning the effect of radium-223 monotherapy on fracture risk. Our data suggest that radium-223 may also increase the risk of fracture when used as a single agent.

The majority of fractures (68%) were at sites of uninvolved bone and not at metastatic sites. This is consistent with results from the ERA 223 trial, in which 78% of fractures were at uninvolved sites.^2^ This suggests that most fractures in these patients are related to poor bone health (fragility fractures), rather than a direct consequence of bone metastases (pathologic fractures). This is supported by the observation that fracture risk was associated with prior use of corticosteroids, which are known to impair bone health.

The fracture rates reported in men with mCRPC receiving radium-223 vary widely from one study to another. In ALSYMPCA, fractures were seen in 4% of patients receiving radium-223 versus 5% receiving placebo.^1^ In ERA 223, fractures were seen in 29% of patients receiving radium-223 versus 11% of patients receiving placebo.^2^ The more frequently imaging is performed, the greater the probability that fractures will be detected. It is not surprising that the fracture rates in ALSYMPCA were relatively low, given that imaging was only done if clinically indicated. We observed fractures in 56% of patients receiving radium-223 in REASURE, even higher than the rate seen in ERA 223. This may reflect the high frequency of previous use of corticosteroids and the low use of bone heath agents in REASURE.

We observed fractures in 33% of our reference cohort of men with bony mCRPC not treated with radium-223. This rate appears relatively high in comparison with previous studies, which may also reflect differences in the use of prior corticosteroids and bone health agents. In the PREVAIL trial, among patients receiving enzalutamide, fractures were seen in 12% compared with 8% for the control arm.^11^ In the COU-AA-301 study, among patients receiving abiraterone and prednisolone, fractures were seen in 21% compared with 8% for the control arm.^12^ In both of these trials, the use of bone health agents (25% in PREVAIL and 45% in COU-AA-301) was higher than in REASURE. Given that the patient characteristics, and particularly the use of bone health agents, vary among studies, we believe that our reference cohort provides the most useful benchmark against which to interpret the risk of fracture in REASURE.

The main strength of REASURE is that it is the first prospective study, to our knowledge, of men treated with radium-223 monotherapy with the use of imaging at prespecified intervals to enable assessment of fracture risk during and after treatment. However, it has several limitations. First, the observed fracture risk may be regarded as a worst-case scenario, given that only a small proportion of patients were receiving a bone health agent, and as many as half had received prior corticosteroids. Second, half of the patients received an escalated dose of radium-223 (88 kBq/kg); however, their fracture risk did not differ from those receiving the standard radium dose (55 kBq/kg). Third, some of the fractures seen on imaging may not be clinically relevant. However, even if, as we found, only half of patients with fractures are symptomatic, the risk of new fracture in mCRPC patients treated with radium-223 remains clinically important. Fourth, regular imaging will result in earlier detection of asymptomatic fractures. This would be true regardless of the imaging modality: MRI (bone edema), bone scan (osteoblastic reaction), or CT (sclerosis). Using bone scans or CT scans, care must be taken to avoid misinterpreting these fractures as new sites of disease. The advantages of WB-MRI are that it distinguishes fractures from disease progression and it allows timely differentiation between a malignant and non-malignant fracture.

The magnitude of the difference in fracture risk between men receiving radium-223 in REASURE and those in the reference cohort should be interpreted with caution. Although the reference cohort was selected to be similar to the REASURE population with regard to major risk factors for fracture (prior corticosteroids, bone health agents), it was not a randomized comparison. The two groups may have differed with respect to other risk factors for fracture, such as age, duration of prior hormone therapy, smoking, and alcohol intake. The frequency of scans in the reference cohort was less than in REASURE, so fractures may also have been detected later in the reference cohort. However, this would not have affected the overall number of fractures detected over the whole observation period.

The study was not designed to assess the impact of bone health agents on fracture risk. However, bone health agents are known to reduce the risk of fracture in men with mCRPC, and it seems clear from the ERA 223 trial and the early results from the PEACE III trial that the use of bone health agents substantially reduces the risk of fracture in men treated with radium-223.^13^ For example, in the radium-223 arm of the ERA 223 trial, fractures were seen in 15% versus 37% of those who were or were not on a bone health agent, respectively.^2^ Taken together with our findings, we believe that it should now be mandatory to use a bone health agent in men treated with mCRPC, particularly those receiving radium-223.

There remains uncertainty as to when bone health agents should be started and what dose schedule should be used. Given that the aim of such treatment is to prevent fragility fractures, we believe that the use of bone heath agents should be similar to that in other populations at risk of fragility fracture, such as postmenopausal women. The European Society for Medical Oncology Clinical Practice Guidelines recommend that men starting long-term androgen deprivation should be offered an oral bisphosphonate or be monitored with dual-energy absorptiometry (DEXA) scanning and be treated according to the bone density results.^14^ Our policy is to recommend the use of an oral bisphosphonate, such as alendronic acid, in men starting long-term androgen deprivation.

In summary, regular imaging with WB-MRI shows that men with mCRPC, and particularly those treated with radium-223, have a high risk of developing new, predominantly non-malignant (osteoporotic) fractures. Men with mCRPC should receive a bone health agent to reduce their risk of fragility fractures.

### Clinical Practice Points

• Radium-223, used in mCRPC, has been shown to increase the risk of fracture when used in combination with abiraterone and prednisolone; however, the risk of fracture in men receiving radium-223 monotherapy is unclear.
• In this prospective study of radium-223, regular imaging assessments were utilized to assess 36 men for fracture incidence. This was matched with 36 men not treated with radium-223 who had a similar fracture risk and also received regular imaging assessments. We identified 74 new fractures in 20 patients (56%) receiving radium-223, and 16 new fractures were identified in 12 patients (33%) not receiving radium-223. Of all fractures across both cohorts, 67% occurred at uninvolved bone (ie, non-pathological fractures).
• Men with mCRPC, particularly those receiving radium-223, are at risk of fracture and should receive a bone health agent to reduce the risk of fragility fractures
